# The Protective Effect of Glycyrrhizic Acid on Renal Tubular Epithelial Cell Injury Induced by High Glucose

**DOI:** 10.3390/ijms150915026

**Published:** 2014-08-26

**Authors:** Shaozhang Hou, Fangfang Zheng, Yuan Li, Ling Gao, Jianzhong Zhang

**Affiliations:** 1 Department of Pathology, Ningxia Medical University, Yinchuan 750004, China; E-Mail: zhangjz@nxmu.edu.cn; 2 Ningxia Key Laboratory of Cardiovascular & Cerebrovascular Diseases, Yinchuan 750004, China; 3 Department of Pharmacy, Ningxia Medical University, Yinchuan 750004, China; E-Mails: xin7998@163.com (F.Z.); gl@nxmu.edu.cn (L.G.); 4 Department of Nursing, Ningxia Medical University, Yinchuan 750004, China; E-Mail: nyliyuan@163.com

**Keywords:** diabetic nephropathy, glycyrrhizic acid, NRK-52E

## Abstract

The aim of this study was to determine the beneficial effect of glycyrrhizic acid (GA) on type 2 diabetic nephropathy using renal tubular epithelial cell line (NRK-52E). The cells are divided into normal group (NG), high glucose group (HG), and treatment group (HG + GA). The methylthiazoletetrazolium (MTT) assay was used to detect the cell proliferation. Cell cycle analysis was performed using flow cytometry. Model driven architecture (MDA), reactive oxygen species (ROS) and superoxide dismutase (SOD) were also measured. Electron microscopy and histological were used to detect the changes in cell ultrastructure. The phosphorylation of AMP-activated protein kinase (AMPK), silent information regulator T1 (SIRT1), manganese-superoxide dismutase (Mn-SOD) and transforming growth factor-β1 (TGF-β1) were assessed by immunohistochemistry, immunofluorescence, and western blotting. Real-time fluorescent quantitative PCR (RT-qPCR) was used to measure Mn-SOD and PPARγ co-activator 1α (PGC-1a) mRNA. We find that high glucose increases NRK-52E cell proliferation and TGF-β1 expression, but decreases expression of AMPK, SIRT1 and Mn-SOD. These effects are significantly attenuated by GA. Our findings suggest that GA has protective effects against high glucose-induced cell proliferation and oxidative stress at least in part by increasing AMPK, SIRT1 and Mn-SOD expression in NRK-52E cells.

## 1. Introduction

Diabetic nephropathy (DN), one of the most severe microvascular complications of type 1 and type 2 diabetes, is a major cause of end-stage renal disease [1]. It seriously affects patients’ life quality. Pathological manifestations of DN include glomerular hypertrophy, basement membrane thickening and accumulation of extracellular matrix, tubulo-interstitial disease, and glomerulosclerosis. The pathogenesis of DN is very complex and has not been fully elucidated. High glucose is the foundation and key of DN, and can induce an abnormal increase of reactive oxygen species (ROS) and oxidative stress occurrence. In addition, high glucose can induce abnormal glucose metabolism and hemodynamic changes. High glucose-induced renal tubular epithelial cells have been used as an *in vitro* model for studies of early-stage DN.

Tubular epithelial cells account for 90% of the total kidney volume. Recent studies showed that the degree of seriousness of tubular interstitial disease is closely related to the DN renal dysfunction. The early-stage DN mainly results in glomerular proteinuria, but its long-term prognosis depends on the severity of tubulointerstitial damage. Tubular epithelial cell hypertrophy is a major factor in causing kidney hypertrophy. Therefore, the early hypertrophy inhibition for diabetic renal tubular epithelial cells helps to control the DN disease.

Functions of tubular epithelial cells can be affected by many external factors such as transforming growth factor-β (TGF-β). In the normal situation, TGF-β can inhibit the cell proliferation and inflammation. However, over-expression of TGF-β may cause pathological changes and promote cell proliferation and extracellular matrix accumulation. The increase of ROS and model driven architecture (MDA) generation, and decrease of antioxidant enzyme superoxide dismutase (SOD) activity are the result of oxidative stress. Studies have shown that high glucose-induced oxidative stress and renal cortical injury is related to down-regulation of PPARγ co-activator 1α (PGC-1α) expression [2].

AMP-activated protein kinase (AMPK) is a serine/threonine kinase evolutionarily conserved with a catalytic α-subunit and regulatory β- and γ-subunits, forming a heterotrimeric complex. It is abundantly expressed in the kidney [3]. AMPK has become a hot research subject for type 2 diabetes. Previous studies have shown that manganese superoxide dismutase (Mn-SOD) can reduce high glucose induced increase of ROS, thereby activate AMPK [4].

Glycyrrhizic acid (GA) is a triterpenesaponin glycoside, which is the primary bioactive component of jor plant root extract of *Glyccyrhiza Glabra* (Liquorice), a shrub from the Leguminosae family [5,6]. Recently, a study showed that GA was able to protect rabbits from renal ischemia reperfusion injuries [7]. Another report shows that after treating diabetic rats with glycyrrhizin for 60 days, TGF-β1 expression in renal tissue was decreased [8].

However, little information is available about the effect of GA on the proliferation of tubular epithelial cells induced by high glucose. The aim of this study was to test the hypothesis if GA has a protective effect against high glucose-induced tubular epithelial cells damage by reducing cell proliferation and oxidative stress. We employed multiple approaches to examine the expression of factors such as AMPK, SIRT1 (silent information regulator T1), Mn-SOD and TGF-β1 in NRK-52E cells in the absence or presence of high glucose, GA, or both. Our data are consistent with our hypothesis.

## 2. Results and Discussion

### 2.1. GA (Glycyrrhizic Acid) Reverses the High Glucose-Induced Effect on Cell Proliferation in NRK-52E Cells

**Figure 1:**
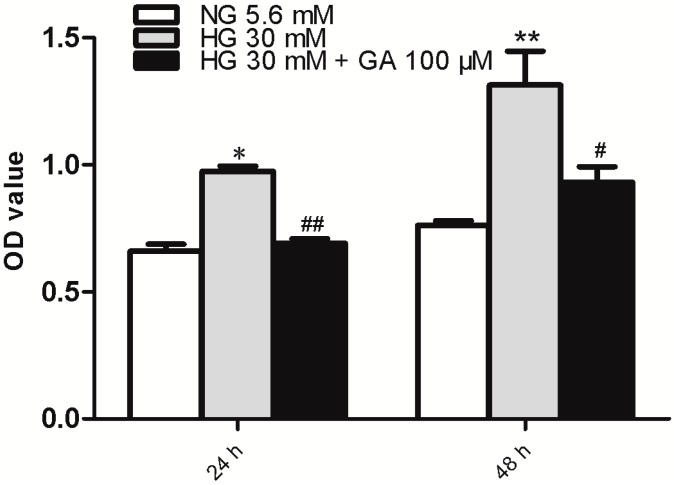
Proliferation assay. NRK-52E cells were treated with high glucose (HG) with or without glycyrrhizic acid (GA) as indicated for 24 or 48 h, followed by MTT (methylthiazoletetrazolium) analyses. Cells receiving normal glucose (NG) were included as control. *n* = 3. * *p* < 0.05 *vs.* NG; ** *p* < 0.01 *vs.* NG; ^#^ *p* < 0.05 *vs.* HG; ^##^ *p* < 0.01 *vs.* HG. OD (optical density).

NRK-52E cell proliferation was evaluated using MTT (methylthiazoletetrazolium) analysis. The results showed that compared with the NG (normal group) group, 30 mM glucose alone increased NRK-52E cell proliferation at both 24 and 48 h time points (*p* < 0.05). We tested the effect of GA at 25, 50, 100, 200 μmol/L and found GA at 100 μmol/L can inhibit NRK-52E cell proliferation induced by HG (*p* < 0.05) (Figure 1).

### 2.2. Effect of GA on Cell Cycle Induced by HG (High Glucose) in NRK-52E Cells

**Figure 2:**
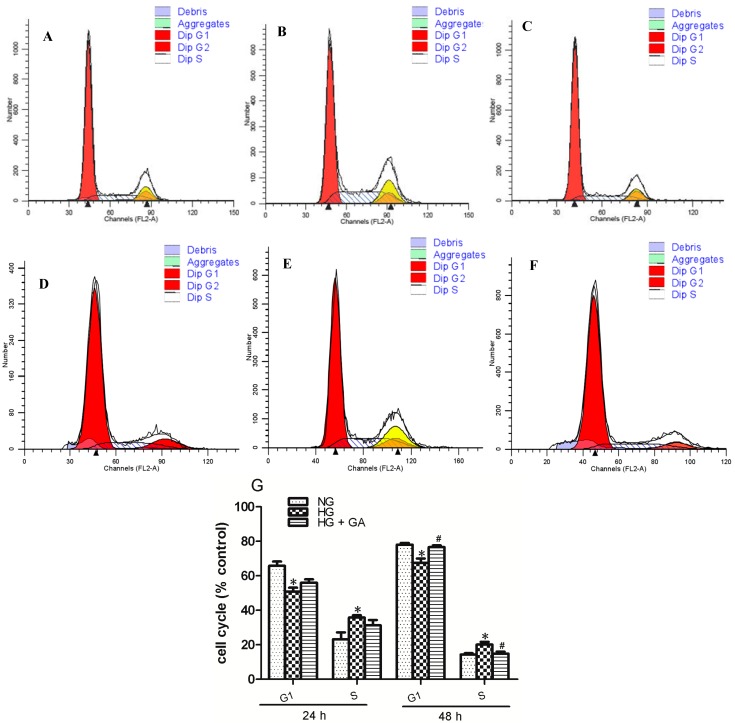
Flow cytometric analysis of the cell cycle. Cells were treated for 24 or 48 h as indicated, (**A**) NG group; (**B**) HG group; (**C**) HG + GA group (treated with 24 h); (**D**) NG group; (**E**) HG group; (**F**) HG + GA group (treated with 48 h); and (**G**) Followed by flow cytometric analysis. * *p* < 0.05 *vs.* NG; ^#^ *p* < 0.05 *vs.* HG.

A flow cytometry was used to evaluate the effect of GA treatment upon cell cycle profiles (Figure 2A–F). After 24 and 48 h incubation in HG group, the proportion of G1 phase decrease and S phase increase in NRK-52E cells (*p* < 0.05). In contrast, more cells in G1 phase and fewer cells in S phases were significantly obtained in GA group after 48 h incubation. (*p* < 0.05). At 24 h time point, GA did not significantly increase the number of cells in G1 (*p* > 0.05), or decrease the cells number in S phase (*p* > 0.05), compared with HG group (Figure 2G).

### 2.3. Histopathological Findings

**Figure 3:**
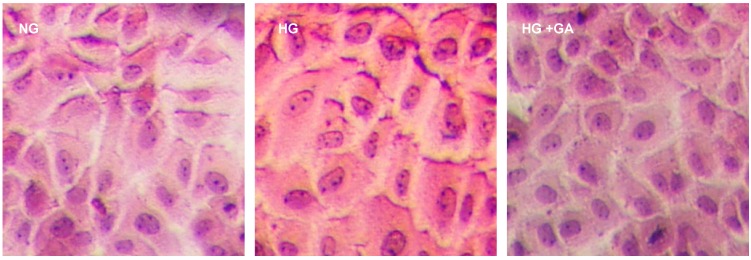
Effect of GA on cell morphology induced by HG in NRK-52E cells. The cells were incubated in the three groups for 24 h. They were stained with hematoxylin-eosin. Original magnification: ×200.

**Figure 4:**
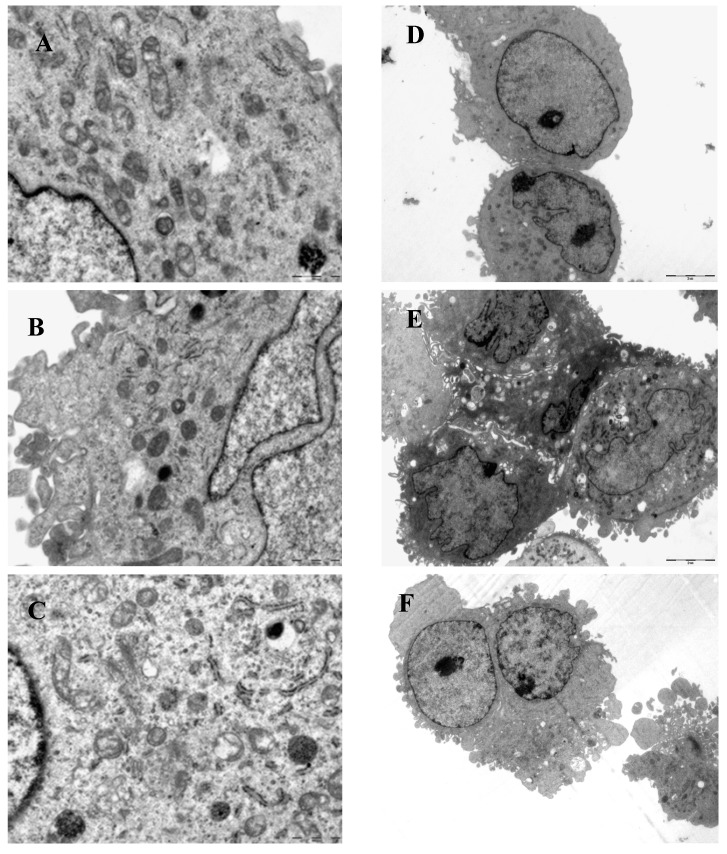
Electron microscopy analyses. NRK-52E cells were grouped and treated as in Figure 1 and examined under an electron microscopy after 48 h. (**A**,**D**) NG group; (**B**,**E**) HG group; (**C**,**F**) HG + GA group. **A**–**C**: ×4000, **D**–**E**: ×1000.

For the NG group, most of NRK-52E cells were round or oval-shaped. Cilia existed in membrane. Organelles were normal. Membrane integrity was good. For the HG group, some cells increased volumes significantly. The number of round cells also increased. Cells tended to change fusiform (Figure 3). The cells incubated in HG exhibited injury features, including an irregular nucleus, chromatin condensation, nuclear envelope shrinkage, organelle shortage, rough endoplasmic reticulum slight expansion, cilias fusion and shortage. The situation became much better in the groups treated with GA (final concentration of 100 μmol/L) in comparison with the HG group (Figure 4).

### 2.4. Effect of GA on SIRT1 (Silent Information Regulator T1), AMPKα (AMP-Activated Protein Kinase α), Mn-SOD (Manganese-Superoxide Dismutase) and TGF-β1 (Transforming Growth Factor-β1) Proteins Expression

**Figure 5:**
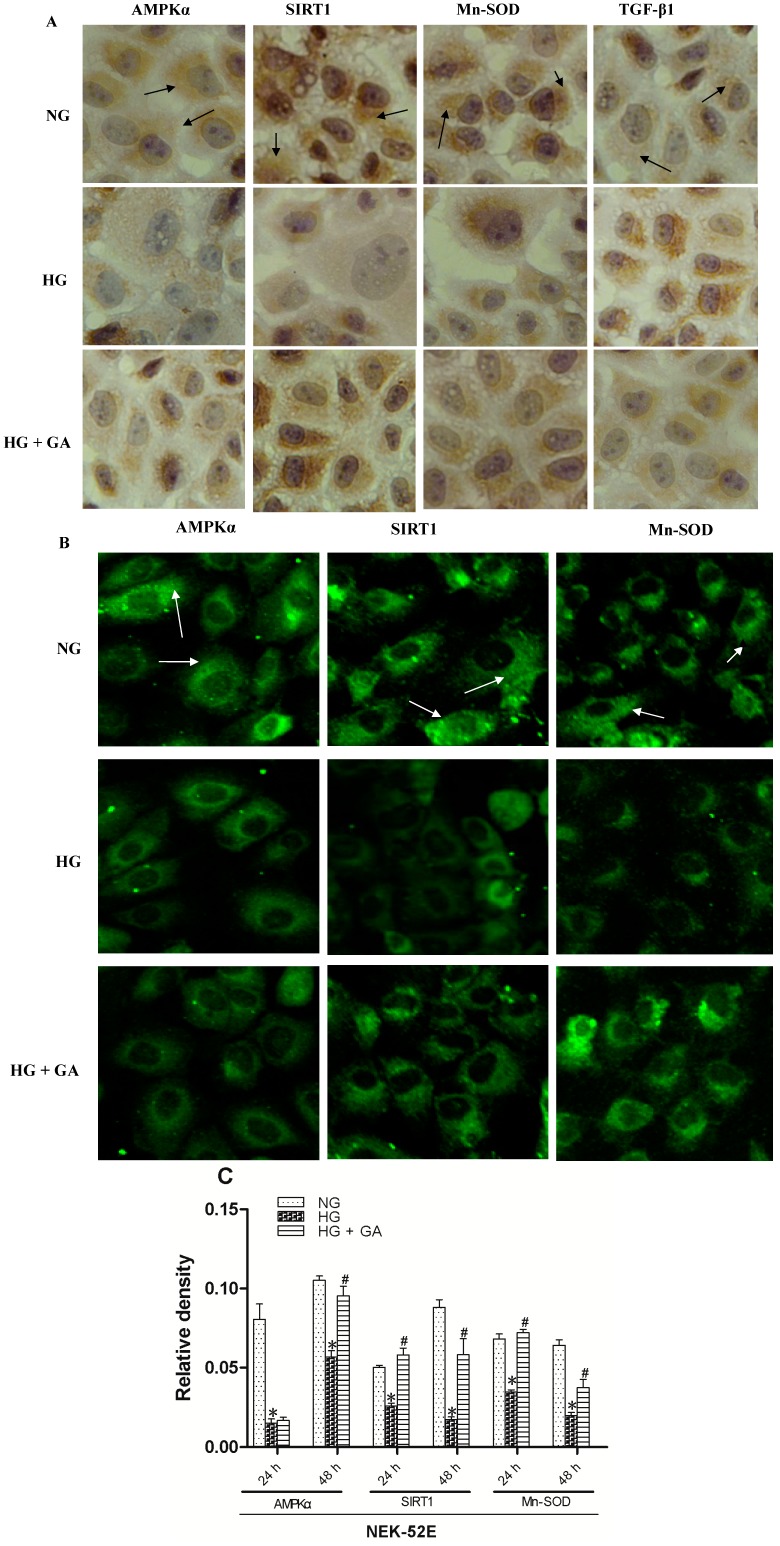
Immunohistochemistry and immunofluorescence analyses. (**A**) NRK-52E cells were grouped and treated as in Figure 1 for 24 h, followed by immunohistochemistry to assess expression of proteins as indicated. Original magnification: ×400; (**B**) As in **B**, except that cells were analyzed by immunofluorescence staining. Original magnification: ×200; and (**C**) Relative levels of protein expression. * *p* < 0.05 *vs.* NG; ^#^ *p* < 0.05 *vs.* HG. Apoptosis is labeled with arrow.

SIRT1, AMPKα and Mn-SOD proteins were detected by immunohistochemistry (Figure 5A and Table 1) and immunofluorescence (Figure 5B,C). TGF-β1 was detected by immunohistochemistry. The results showed that the fluorescence intensity and optical density in the HG group were lower than that in the control group for SIRT1, AMPKα and Mn-SOD (*p* < 0.05). SIRT1 and Mn-SOD increased in the GA group (*p* < 0.05). However, for AMPKα, it increased only at 48 h but not at 24 h (*p* > 0.05). Immunohistochemical experiment showed that HG increased TGF-β1 protein expression, which was reversed in the GA group (*p* < 0.05).

**Table 1:** Effect of GA on silent information regulator T1 (SIRT1), AMP-activated protein kinase α (AMPKα), manganese-superoxide dismutase (Mn-SOD) and transforming growth factor-β (TGF-β1) proteins expression in NRK-52E cells, as detected by immunohistochemistry.

Group (*n* = 6)	AMPKα Expression	SIRT1 Expression	Mn-SOD Expression	TGF-β1 Expression
Normal group (24 h)	0.24 ± 0.018	0.21 ± 0.014	0.27 ± 0.018	0.17 ± 0.006
High glucose group (24 h)	0.20 ± 0.027 *	0.18 ± 0.015 *	0.19 ± 0.031 *	0.28 ± 0.019 **
Experimental group (24 h)	0.22 ± 0.025	0.21 ± 0.017 ^#^	0.24 ± 0.028 ^#^	0.18 ± 0.017 ^##^
Normal group (48 h)	0.29 ± 0.010	0.19 ± 0.017	0.41 ± 0.048	0.23 ± 0.005
High glucose group (48 h)	0.23 ± 0.007 **	0.13 ± 0.006 *	0.22 ± 0.029 *	0.31 ± 0.058 **
Experimental group (48 h)	0.25 ± 0.006 ^#^	0.18 ± 0.005 ^#^	0.28 ± 0.024 ^#^	0.26 ± 0.037 ^#^

* *p* < 0.05 *vs.* NG; ** *p* < 0.01 *vs.* NG; ^#^ *p* < 0.05 *vs.* HG; ^##^ *p* < 0.01 *vs.* HG.

### 2.5. Effect of GA on SIRT1, AMPKα, Mn-SOD and TGF-β1 Proteins Expression, as Detected with Western Blotting

**Figure 6:**
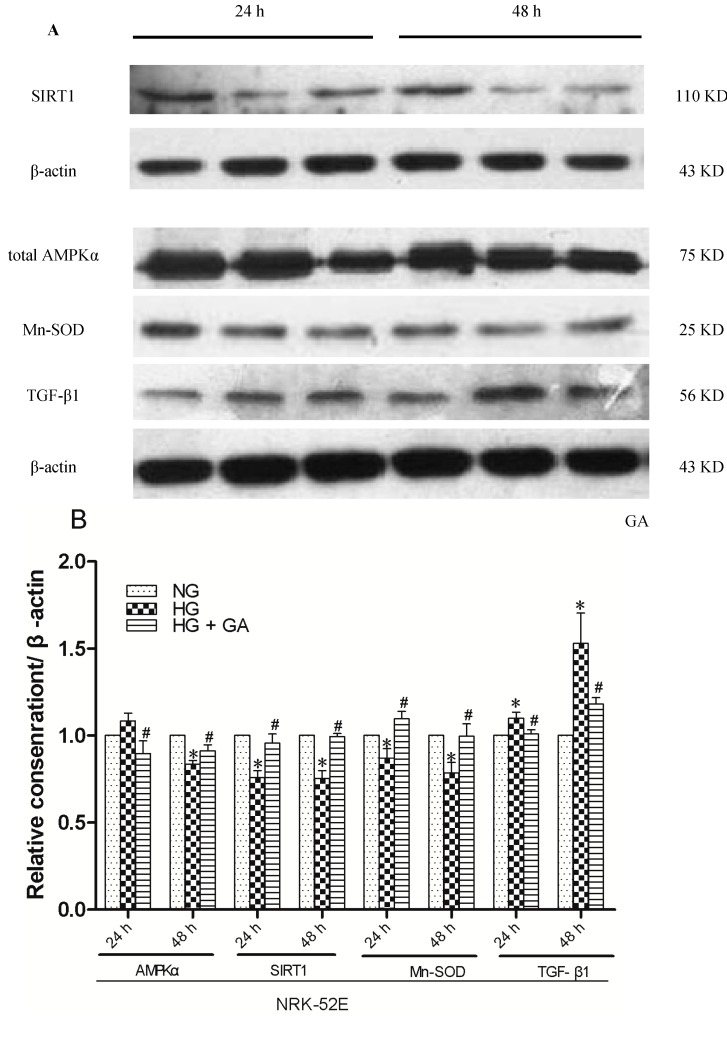
Western blotting analyses. NRK-52E cells were grouped and treated as in Figure 1 for 24 and 48 h, followed by Western blotting to evaluate expression of proteins as indicated, with Actin as an equal loading control. (**A**) Western blot detect purpose proteins treated with 24 and 48 h pictures; (**B**) Relative levels of protein expression. The intensity of the bands on Western blots were quantified, normalized to Actin, and graphed. * *p* < 0.05 *vs.* NG; ^#^ *p* < 0.05 *vs.* HG.

In our experiment, there was a decrease in the expression of AMPKα, SIRT1 and Mn-SOD, and an increase in the expression of the TGF-β1 after treatment of cells with HG. However, the treatment with GA antagonized all the above effects induced by HG (Figure 6A,B).

### 2.6. Effects of GA on Activities of Antioxidant Enzymes and Oxidative Stress Markers

The activity of SOD and concentration of MDA were lower, whereas concentration of MDA was higher in the HG group than in the control group (*p* < 0.05), suggesting that the cells were suffered from oxidative stress (Table 2). Treatment with GA significantly decreased the concentrations of MDA and significantly increased SOD activity (*p* < 0.05). These results indicate that GA ameliorates oxidative stress in high glucose cells.

**Table 2:** Effect of GA on model driven architecture (MDA) and SOD in NRK-52E cells.

Group (*n* = 3)	MDA (μmol/L)	SOD (U/mL)
Normal group	7.57 ± 0.680	23.60 ± 0.538
High glucose group	14.66 ± 0.480 **	16.22 ± 0.315 **
Experimental group	9.77 ± 0.468 ^##^	19.68 ± 0.952 ^#^

** *p* < 0.01 *vs.* NG; ^#^ *p* < 0.05 *vs.* HG; ^##^ *p* < 0.01 *vs.* HG.

### 2.7. Effect of High Glucose and GA on ROS (Reactive Oxygen Species) Production

**Figure 7:**
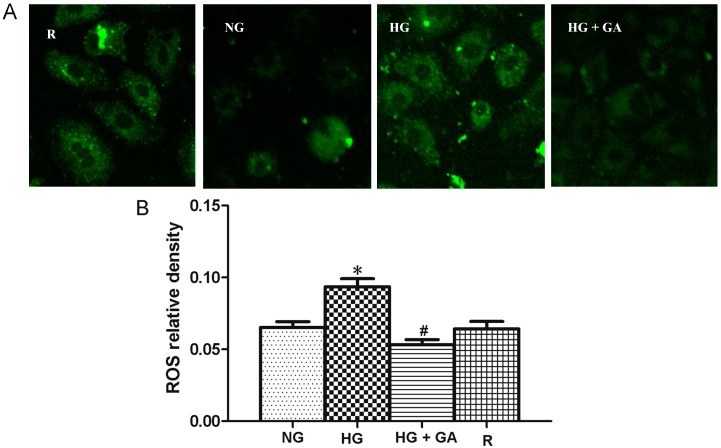
High glucose induces ROS (reactive oxygen species). Cells were grouped and treated for 24 h as indicated, followed by DCFH-DA (2',7'-dichlorofluorescin diacetate) (probe) or rosup (positive control) treatment for 30 min. Cell were then analyzed, using confocal microscopy. R represents rosup. (**A**) confocal microscopy images; (**B**) Histograms. * *p* < 0.05 *vs.* NG; ^#^ *p* < 0.05 *vs.* HG.

We observed that the ROS production was augmented in NRK-52E cells induced with high glucose, peaking after 24 h of treatment (Figure 7A). With the addition of GA, it was decreased. These data indicate that after giving GA, there appears either a decrease in ROS generation or an increase in endogenous ROS scavenging/antioxidant capacity.

### 2.8. Effect of GA on the Expression of Mn-SOD and PGC-lα (PPARγ Co-Activator 1α) mRNA

**Figure 8:**
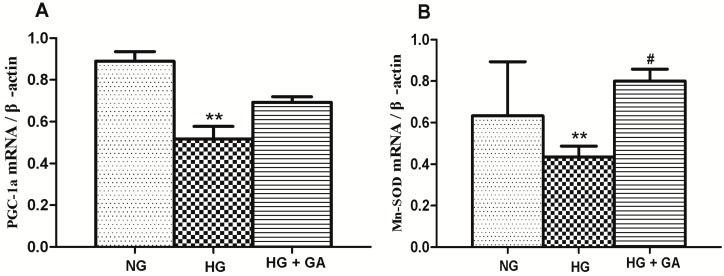
Real-time RT-qPCR analyses. Effect of GA on Mn-SOD (**A**) and PGC-1α (PPARγ co-activator 1α) (**B**) mRNA expression was measured by real-time fluorescent quantitative RT-qPCR. Values were normalized to mouse housekeeping gene β-actin and then normalized to sham samples. The mRNA levels of Mn-SOD and PGC-1α in the HG group were significantly lower than those in the NG group (** *p* < 0.01). The mRNA levels of Mn-SOD began to return to higher levels in the GA group (^#^ *p* < 0.01). But no change was observed in PGC-lα mRNA level for GA group compared with HG group.

The effects of GA on Mn-SOD and PGC-lα mRNA in NRK-52E cells have been examined. Exposure of NRK-52E decreased the expression of Mn-SOD and PGC-lα mRNA level in the HG group compared with NG group. The expression of Mn-SOD increased in GA group (*p* < 0.05), but PGC-lα mRNA level did not increase in GA group (Figure 8A,B) (*p* > 0.05).

### 2.9. Discussion

Previous studies have shown that high glucose concentrations promote proliferation of mesangial cells, renal tubular epithelial cells, and vascular smooth muscle cells under the diabetic state [9]. In this study, 30 mmol/L high glucose was used as the stimulator to induce NRK-52E cell proliferation. Rat renal tubular epithelial cells induced by high glucose as an *in vitro* model have been used in studies of early-stage diabetic nephropathy. Clinical trials have shown that high glucose is the principal cause of renal damage in both type 1and type 2 diabetes [10]. MTT is a method to detect cell proliferation activity and was used in this study to detect the NRK-52E cell proliferation. The results shows that high glucose significantly increases NRK-52E cell proliferation after intervention for 24 and 48 h. This demonstrated that NRK-52E cells can be used as *in vitro* model of diabetic nephropathy after injury through induction with 30 mmol/L high glucose. It was also shown that GA can inhibit the cell proliferation induced by HG at the concentration of 100 µmol/L.

Reponses of mesangial cells to injuring stimuli include proliferation, hypertrophy and apoptosis. The degree of these injuries depends on the progress of the cell cycle. In cell cycle progression, traversing the G1-S phase boundary is coupled to DNA synthesis. In vitro, high glucose can promote mesangial cells from G1 to S phase, reducing the proportion of cells in G1 phase, and increasing the proportion of cells in S phase. The high glucose also causes DNA synthesis and promotes cell proliferation [11]. It was shown that GA can inhibit DNA synthesis, resulting in arrest of the cell-cycle transition from G1 to S phase. Compared with NG group, high glucose significantly increased the NRK-52E cells population at S-phase, whereas 100 μmol/L GA blocked the G1-S phase transition after the cells were treated for 48 h. GA may be involved in inhibiting cell proliferation.

These results suggest that GA inhibits high glucose-induced mesangial cell proliferation to execute its protective effect on NRK-52E cell injury.

Although the mechanisms of proliferation of NRK-52E cells induced by high glucose are still poorly defined, mechanism of ROS is well documented in both diabetes and model systems. In addition, high glucose has been frequently shown to augment cell proliferation and DNA synthesis, and it has been suggested as the main contributor to DN in affected subjects [12]. The importance of elevated ROS level in the pathogenesis of the diabetes-related microvascular complications has been well documented [12,13]. High glucose-induced increase of ROS is an important aspect of oxidative stress. The results shows that ROS increased in HG group compared with the NG group. However, it was decreased in GA group compared with the HG group.

The cellular levels of ROS and the presence of oxidative stress are determined not only by rates of ROS generation, but also by their neutralization and degradation using endogenous antioxidants. Oxidative stress leads to lipid peroxidation and thereby formation of the harmful product such as MDA [14]. Accordingly, it induces DNA oxidative damage via generation of 8-OHdG (8-hydroxy-2-deoxy Guanosine) [15]. Meanwhile, the activity of antioxidant defense enzymes decreases [16], which leads to cell damage. The reduced activity changes the oxidative stress markers: decreasing SOD levels and increasing MDA. It was reported that an anti-oxidative role of GA in the amelioration of carbon tetrachloride-induced liver injury [17].

The peroxisome PGC-1α is a small family of transcriptional coactivators, which play a critical role in the control of glucose, lipid, and energy metabolism [18]. The physiological significance of PGC-1α in mitochondrial energy metabolism has been well demonstrated [19,20]. Studies have shown that high glucose-induced renal cortical injury and oxidative stress is related to down-regulation of PGC-1α. When PGC-1α stimulates mitochondrial biogenesis, SOD generation increases [21]. Therefore, when PGC-1α down-regulates, oxidation and antioxidant system become imbalanced and oxidative stress occurs. In this experiment, we used RT-qPCR method to detect the PGC-1α mRNA. The result showed that compared with NG group, PGC-lα mRNA decreased in HG group. There was no different in GA group compared with HG group.

These results showed that high glucose can promote ROS generation, decrease SOD and increase MDA in NRK-52E cells. It was further confirmed that high glucose can induce ROS production, promote lipid peroxidation, and decrease antioxidant capacity. In GA group, however, it was observed that GA had an antioxidant role, ROS, causing MDA to decrease and SOD to increase. We also used RT-qPCR method to detect the Mn-SOD mRNA. The result showed that Mn-SOD mRNA increase in GA group. These results suggest that GA can reduce the oxidative stress, improve antioxidant system to protect cells from damage. However, the protective effect of glycyrrhizin on high glucose-induced NRK-52E cells’ oxidative stress injury may not be regulated by PGC-1α after treatment for 24 h.

Since AMPK is a cellular energy sensor, its activity is highly linked to the change in the intracellular AMP/ATP ratio. The role of AMPK in carbohydrate and protein metabolism, cell cycle regulation, and in mitochondrial biogenesis has been described in literature. AMPK also regulates glucose homeostasis. Insulin deficiency has been proposed as one of the factors causing hypothalamic. AMPK activation and the subsequent increase in food intake have been observed in streptozotocin-induced diabetic rats [22]. Renal AMPK activity was reduced after a high-fat diet (HFD) for 16 weeks [23]. However, its role in mediating renal inflammation was rarely evaluated. Adiponectin and rosiglitazone can activates AMPK through improving insulin sensitivity. We used immunohistochemistry, immunofluorescence and western blot experiments to detect AMPK protein expression. The results showed that AMPKα has a wealth expression in the NRK-52E cells. AMPKα protein expression decreased in high glucose group. GA could make AMPKα protein expression increased after treatment for 48 h. It also showed that AMPK was localized in the cytoplasm.

Sirt1, a NAD-dependent protein deacetylase, is reported to regulate intracellular metabolism and attenuate ROS-induced apoptosis, leading to longevity and acute stress resistance. Both AMPK and SIRT1 have emerged as interesting targets as they are heavily involved in catabolic metabolism, mitochondrial activation, angiogenesis and enhanced cell survival [24,25,26,27]. It is well known that the effects of resveratrol are mediated by both SIRT1 and AMPK [28]. However, the direct relationship between kidney-specific Sirt1 and renal tissue survival *in vivo* has not been elucidated. In this study, immunohistochemistry, immunofluorescence and western blot experiment showed that high glucose can reduce the expression of SIRT1, which may be attentuated after giving GA.

TGF-β1 is a key intermediary substance in DN. TGF-β1 can form an autocrine activation of proliferation ring in an autocrine manner to prevent glomerular mesangial cells, epithelial, endothelial cells and proximal tubule cell from proliferation, differentiation and increasing cell diameter. In early diabetic, glomerular and tubular hypertrophy plays an important role. The experimental showed that TGF-β1 protein expression was significantly increased in HG group after 24 and 48 h, which may be reduced after giving GA. Morphology shows that cells were flat in HG group compared with NG group. In the electron microscope after 48 h treatment, stimulation showed that NRK-52E cell’s volume increased comparing to NG group. This suggests that cells hypertrophy in HG group relates to high expression of TGF-β1.

These results suggest that GA protects NRK-52E cells from damage through regulating the expression of AMPK, SIRT1, Mn-SOD and TGF-β1. NRK-52E cells hypertrophy induced by high glucose correlates to high expression of TGF-β1.

## 3. Experimental Section

### 3.1. Materials

Cell culture reagents, trypsin EDTA (Calcium disodium), fetal bovine serum (FBS) were obtained from Hyclone laboratories Inc. (South Logan, UT, USA). Flasks, vials, 24 Well cell culture cluster, 96 well cell culture cluster and straws were purchased from Corning Incorporated (Corning, NY, USA). Glycyrrhizin was come from Tokyo Chemical Industry Co. (TCI, Tokyo, Japan). d-(+)-glucose powder was purchased from Sigma (St. Louis, MI, USA). NRK-52E cells were obtained from Chinese Center for Type Culture Collection (Shanghai, China). MTT (5 mg/mL) and cell cycle detection kits were purchased from Keygen Biotech (Nanjing, China). Reaction Oxygen Species Assay Kit was purchased from Beyotime (Shanghai, China). SOD and MDA kits were obrained from Nanjing Jiancheng (Nanjing, China). The antibodies recognizing AMPKα, SIRT1 and Mn-SOD were purchased from Santa Cruz (TX, USA). Anti-β-actin was purchased from Bios (Beijing, China). Trizol was purchased from Invitrogen (Grand Island, NY, USA). Other commercial kits of the Transcript RT/RI Enzyme Mix and the TransStart Top Green qPCR SuperMix were obtained from TransGen Biotech (Beijing, China). All other reagents were from commercial sources and of standard biochemical quality.

### 3.2. Cell Culture

The cells were cultured in DMEM (Dulbecco’s modi-fied Eagle’s medium) supplemented with 10% fetal calf serum, 100 U/mL penicillin, and 100 μg/mL streptomycin at 37 °C in an atmosphere containing 5% CO_2_. After preincubation in DMEM supplemented with 0.5% fetal calf serum for 24 h, cells were then treated in three different groups: normal glucose group (NG, 5.6 mM glucose), high glucose group (HG, 30 mM glucose), and high glucose and glycyrrhizic acid group. Cells were incubated for another 24 or 48 h before analyses.

### 3.3. Cell Proliferation Assay

MTT assay was used to measure cell proliferation. After 24 and 48 h incubation with different compounds as described above, 50 μL MTT was added and cells were cultured for additional 4 h. Subsequently, cells were lysed using dimethylsulfoxide. When the formanzan crystals were completely dissolved, the density (OD) was measured at 490 nm, using a Spectrophot-ometer Microplate Reader come from BioTek (Winooski, VT, USA).

### 3.4. Flow Cytometry

After 24 or 48 h treatment with different compounds, cells were harvested by trypsinization without EDTA. They were then centrifuged (with 1000× *g*, 10 min, 4 °C) and washed twice with phosphate buffered saline (PBS). Then they were fixed in methanol at 4 °C overnight. Following two washes with PBS, the fixed cells were incubated in RNase at 37 °C for 30 min, followed by a DNA staining with propidium iodide at 4 °C for 30 min in the dark. Then each sample was analyzed using a Flow Cytometer (BD FACSCalibur, Biocompare, San Diego, CA, USA) and the proportion of cells within the G1 and S phases of the cell cycle were determined.

### 3.5. Histological Studies

After cells were intervened 24 h, the cells were fixed in 95% ethanol. Then stained with hematoxylin-eosin. The stained sections were examined and photographed with a light microscope (BX61, Olympus, Tokyo, Japan).

### 3.6. Electron Microscopy

After cells were intervened 48 h, they were fixed with 4 °C, then washing with 0.1 mol cacodylate buffer. Experimental procedure includes before fixation, after fixation, dehydration, embedding, slicing and uranyl acetate dye staining. TEM (transmission electron microscopic) comes from H7650 (Hitachi, Shiga, Japan).

### 3.7. Immunohistochemistry Assay

Cells were incubated with antibodies specific for AMPKα, SIRT1, Mn-SOD (1:200) and TGF-β1 (1:100, BOSTER, Wuhan China) overnight, followed by secondary antibody incubation in IHC Detection Reagent (ZSGB-BIO, Beijing, China) in 37 °C for 45 min. DAB (ZSGB-BIO) was used to stain cytoplasm. Then hematoxylin was used to stain nucleus. Cells were then examined and photographed with light microscope (BX61). Ipwin32 software was used to quantify optical density.

### 3.8. Immunofluorescence Assay

The cells were cultured on glass coverslips and were treated for 24 and 48 h after growth arrest. Cells were fixed in 4.0% formaldehyde, then blocked with goat serum. Immunofluorescence staining was performed by incubating the fixed cells with anti-AMPKα, SIRT1 and Mn-SOD antibodies (1:200), followed by incubation with FITC (fluorescein isothiocyanate)-conjugated secondary antibody (1:40, Protein Tech Group, Chicago, IL, USA). Cells were viewed with an Olympus FV1000 Laser Scanning Confocal Microscope (Olympus, Center Valley, PA, America). Ipwin32 software (Media Cybernetics Inc., Bethesda, MD, USA) was used to quantify fluorescence intensity.

### 3.9. Western Blotting

After being treated with different compounds for 24, 48 h, cells were harvested and washed with ice-cold phosphate buffer. Protein was obtained using whole-cell extraction kit (Keygen biotech, Beijing, China). Protein concentrations were determined using the BCA method (Keygen biotech). Equal amounts of protein were loaded, separated by SDS-PAGE (SDS-polyacrylamide gel) and transferred to nitrocellulose membranes. After being blocked with 5% skimmed milk in Tris-phosphate buffered saline (TPBS) at room temperature, the membranes were incubated overnight at 4 °C with primary antibodies for AMPKα, SIRT1, Mn-SOD (1:300), TGF-β1 (1:100), and Rabbit An-β-Actin (1:1000). After being incubated with the respective second antibody, immune complexes were detected using ECL (chemiluminescent agent) (Amersham Life Science, Arlington Heights, IL, USA) Western blotting reagents. Immunoreactive bands were quantified using the Bio-RAD Gel Imaging System (Bio-RAD, Berkeley, CA, USA). Values were corrected with the internal control (β-actin).

### 3.10. UV Spectrophotometer

After cells were cultured for 24 h, UV spectrophotomete (VIS-7220N, Beijing, China) was used to detect OD of SOD and MDA between different groups.

### 3.11. ROS Detection

The cells were cultured on glass coverslips and incubated in the dark with 10 μM/L of DCF (dichlorofluorescein) or Rosup for 30 min at 37 °C. Intracellular ROS production was assessed with an Olympus FluoView 1000 Laser Scanning Confocal Microscope (OLYMPUS) (using ex/em λ = 488 nm/515 nm for DCFH-DN).

### 3.12. Quantitative Real Time Polymerase Chain Reaction PCR Assay

RNA was extracted using the Trizol reagent, reversely transcribed with an RT kit using oligo (dT) 18 primer (0.5 μg/μL) in a total volume of 20 μL according to Manufacturer**^’^**s protocol. Real-time PCR was performed and analyzed on a Fluorescent PCR instrument (IQ-5) using cDNA and SYBR Green PCR Master Mix. The primers used were: Mn-SOD forward: 5'-AAGGAGCAAGGTCGCTTACAGA-3', Mn-SOD reverse: 5'-CAAATGGCTTTCAGATAGTCAGGTC-3', PGC-1α forward: 5'-AATCAAGCCACTACAGACACCGC-3', PGC-1α reverse: 5'-CTTTCGTGCTCATTGGCTTCAT-3', The relative amounts of mRNA were determined by the 2^−ΔΔ*C*t^ calculations.

### 3.13. Statistical Analysis

All quantitative data are expressed as mean ± SD. The differences between two experimental conditions were analyzed using the Student’s *t* tests and/or one-way analysis of variance (ANOVA). *p* < 0.05 was considered statistically significant.

## 4. Conclusions

We concluded that the GA treatment resulted in protective effects on NEK-52E cell injuries induced by high glucose. The protection is obtained by inhibiting the high-glucose induced up-regulation of oxidation factor ROS and MDA, and down-regulation of SOD. However, the protective effect of glycyrrhizin on high glucose-induced NRK-52E cells’ oxidative stress injury may not be regulated by PGC-1α. In addition, GA may increase AMPK, SIRT1 and Mn-SOD proteins expression and decreased expression of TGF-β1 induced by HG. NRK-52E cells hypertrophy induced by high glucose may relate to high expression of TGF-β1. At the cell level, GA may be a potential therapeutic agent for the early stage of DN.
